# Exploitation of the Large‐Area Basal Plane of MoS_2_ and Preparation of Bifunctional Catalysts through On‐Surface Self‐Assembly

**DOI:** 10.1002/advs.201700356

**Published:** 2017-09-23

**Authors:** Yinghe Zhao, Qiang Li, Li Shi, Jinlan Wang

**Affiliations:** ^1^ School of Physics Southeast University Nanjing 211189 China; ^2^ Synergetic Innovation Center for Quantum Effects and Applications (SICQEA) Hunan Normal University Changsha 410081 China

**Keywords:** hydrogen evolution reaction, MoS_2_, on‐surface self‐assembly, overall water splitting, oxygen evolution reaction

## Abstract

The development of nonprecious electrochemical catalysts for water splitting is a key step to achieve a sustainable energy supply for the future. Molybdenum disulfide (MoS_2_) has been extensively studied as a promising low‐cost catalyst for hydrogen evolution reaction (HER), whereas HER is only catalyzed at the edge for pristine MoS_2_, leaving a large area of basal plane useless. Herein, on‐surface self‐assembly is demonstrated to be an effective, facile, and damage‐free method to take full advantage of the large ratio surface of MoS_2_ for HER by using multiscale simulations. It is found that as supplement of edge sites of MoS_2_, on‐MoS_2_ M(abt)_2_ (M = Ni, Co; abt = 2‐aminobenzenethiolate) owns high HER activity, and the self‐assembled M(abt)_2_ monolayers on MoS_2_ can be obtained through a simple liquid‐deposition method. More importantly, on‐surface self‐assembly provides potential application for overall water splitting once the self‐assembled systems prove to be of both HER and oxygen evolution reaction activities, for example, on‐MoS_2_ Co(abt)_2_. This work opens up a new and promising avenue (on‐surface self‐assembly) toward the full exploitation of the basal plane of MoS_2_ for HER and the preparation of bifunctional catalysts for overall water splitting.

## 1 Introduction

Developing clean energy has been an irreversible momentum due to the gradual depletion of fossil fuel and the increasing release of carbon dioxide.^1^ Molecular hydrogen produced from electrochemical water splitting has been regarded as one of the cleanest energy carriers because of its nonpollution during the production and the combustion processes.^2^ Platinum (Pt) and its alloy are the most efficient electrochemical catalysts for hydrogen evolution reaction (HER) at present, whereas its high cost hinders the practical application severely.^3^, ^4^ Much effort has been devoted to developing nonprecious materials to replace Pt as highly active catalysts for HER.

With the emergence of 2D materials,^5^, ^6^, ^7^, ^8^, ^9^ several 2D materials have been demonstrated to be of high HER activity, such as MXenes,^10^, ^11^, ^12^, ^13^ C_3_N_4_,^14^, ^15^ boron monolayers,^16^ and molybdenum disulfide (MoS_2_).^17^, ^18^, ^19^, ^20^, ^21^, ^22^, ^23^, ^24^, ^25^, ^26^, ^27^, ^28^, ^29^ Among them, MoS_2_ is the most studied and is identified as the most promising alternative to Pt. Unfortunately, the active sites of pristine MoS_2_ are merely located at the edge, leaving a large area of in‐plane domains useless.^17^, ^19^, ^29^ Defect engineering of MoS_2_ is a common strategy toward the utilization of the basal plane,^20^, ^23^, ^24^, ^25^, ^26^, ^27^, ^28^ whereas it has a side effect on the stability of MoS_2_.^20^, ^27^ Another strategy is phase‐transition engineering of MoS_2_ from 2H to 1T (1T′) phases,^18^, ^21^, ^22^, ^24^ but the metastable nature^21^ of 1T or 1T'‐MoS_2_ hinders its practical applications.^30^, ^31^ Therefore, developing simple, effective, and damage‐free methods to exploit the large‐area basal plane of MoS_2_ is highly demanding.

Recently, molecular self‐assembly on gold and carbon‐based materials has been successfully used to prepare electrochemical catalysts toward O_2_ and CO_2_ reductions,^32^, ^33^, ^34^, ^35^ and 2D transition metal dichalcogenides were demonstrated to be suitable substrates for molecular self‐assembly.^36^ The organic molecules are physically self‐assembled on the underlying substrates via intermolecular weak interactions, thereby leading to no structural damage on the substrates. Inspired by such research, the motivation for the present work is to explore whether the basal plane of MoS_2_ can be fully exploited for HER through on‐surface self‐assembly. To this end, we need to verify that the following two conditions can be satisfied: (i) on‐MoS_2_ molecule possesses high HER activity; and (ii) the molecules self‐assembled on the basal plane of MoS_2_ via intermolecular weak interactions are stable and will not diffuse into water. By means of multiscale simulations combined density functional theory (DFT) and classical molecular dynamics (MD), we demonstrate that both the two preconditions can be fulfilled by M(abt)_2_ (M = Ni, Co; abt = 2‐aminobenzenethiolate) on MoS_2_, showing that on‐surface self‐assembly is indeed an effective approach to take advantage of the large‐area basal plane of MoS_2_ for HER without damage on MoS_2_. Also, a facile method toward the preparation of self‐assembled M(abt)_2_ monolayers is proposed, that is dropping M(abt)_2_ solution onto MoS_2_.

**Figure 1:**
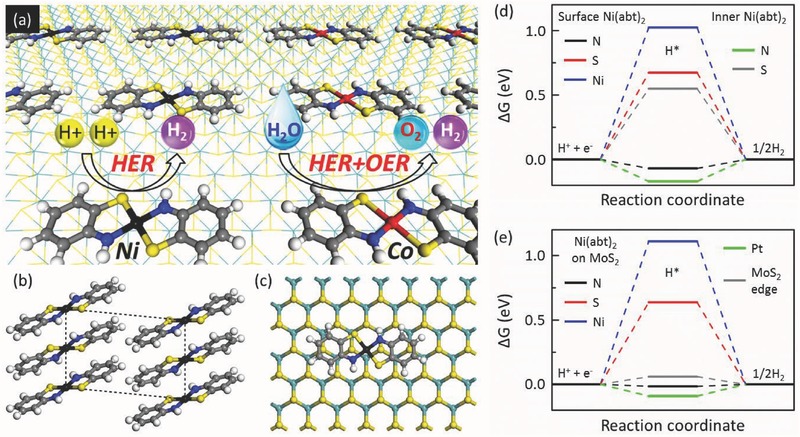
a) Schematic used to reflect our idea. Structures of b) Ni(abt)_2_ crystal and c) Ni(abt)_2_ on MoS_2_. d) Calculated Δ*G* profile of HER at the surface N, S, and Ni sites as well as the inner N and S sites of Ni(abt)_2_ crystal. e) Calculated Δ*G* profile of HER at N, S, and Ni sites of on‐MoS_2_ Ni(abt)_2_. Black, red, gray, white, blue, yellow, and green balls stand for Ni, Co, C, H, N, S, and Mo atoms, respectively.

On‐surface self‐assembly has been used to prepare single‐function catalysts.^32^, ^33^, ^34^, ^35^ However, to the best of our knowledge, the preparation of bifunctional catalysts has never been reported through on‐surface self‐assembly. The rational design and the facile preparation of bifunctional catalysts toward overall water splitting have attracted ever‐increasing attention in recent years because of the reduced production cost compared with two separate single‐function catalysts for HER and oxygen evolution reaction (OER).^37^, ^38^, ^39^, ^40^, ^41^, ^42^, ^43^, ^44^ Our studies show that as complementary to edge sites of MoS_2_ and N sites of on‐MoS_2_ Co(abt)_2_ for HER, OER can be catalyzed at Co sites of on‐MoS_2_ Co(abt)_2_, suggesting that on‐surface self‐assembly is also able to serve for overall water splitting. Our idea, via on‐surface self‐assembly to make the best use of the large‐area in‐plane domains of MoS_2_ for HER and prepare bifunctional catalysts for overall water splitting, is schematically displayed in **Figure** **1**a.

## 2 Results and Discussion

### 2.1 HER Activity of On‐MoS_2_ Ni(abt)_2_

Ni(abt)_2_ crystal has been demonstrated to be of high HER activity experimentally,^45^ and the structure is shown in Figure 1b. We first explore the active sites of the bulk Ni(abt)_2_, including the surface and inner positions of the crystal. The HER and OER activities are both evaluated by the binding free energy (Δ*G*) that was developed by Nørskov and co‐workers,^46^, ^47^ which has proven a powerful way to predict new electrocatalysts and understand the reaction mechanisms.^10^, ^11^, ^14^, ^27^, ^46^, ^47^, ^48^, ^49^, ^50^, ^51^, ^52^ The HER is calculated in acidic circumstance and the process is described as

(1) $$H^{+}\;+\;e^{-}\;+\;*\;⇌\;*H$$

in which the * stands for the catalytic site and the smaller the absolute value of Δ*G* is, the better HER performance is. On the surface, the Δ*G* of N, S, and Ni sites are −0.07, 0.67, and 1.02 eV, respectively (see Figure 1d). It is obvious that the HER activity of N site is far higher than that of S and Ni sites, in good agreement with the experiment.^45^ For the inner part, the N site (Δ*G* is −0.17 eV) also owns better HER activity in comparison with the S site (Δ*G* is 0.55 eV), while the Ni site is unfavorable for hydrogen adsorption due to easy migration to adjacent sites. By comparing Δ*G* of surface and inner N sites, it can be derived that the surface N site is superior to the inner of the bulk material in the HER activity.

In addition, Figure S1d of the Supporting Information records the water distribution when Ni(abt)_2_ crystal is placed in water. The simulation shows that water molecules distribute on the surface of Ni(abt)_2_ crystal and do not penetrate into the inner part of the bulk. Considering that the inner HER activity is not high and water molecules are difficult to diffuse into the bulk, it can be concluded that HER mainly concentrates on the surface N sites of Ni(abt)_2_ crystal. In this respect, it will be highly material‐saving if Ni(abt)_2_ molecules are processed into ultrathin films or even monolayer, which can be potentially realized by bottom‐up on‐surface self‐assembly.^53^, ^54^, ^55^, ^56^, ^57^

Next, Ni(abt)_2_ molecule is placed on the surface of MoS_2_, and the HER activity of on‐MoS_2_ Ni(abt)_2_ is evaluated accordingly. As shown in Figure 1e, Δ*G* of N, S, and Ni sites of on‐MoS_2_ Ni(abt)_2_ are −0.02, 0.64, and 1.11 eV, respectively, and the N site is responsible for HER. The Δ*G* values are similar to the surface sites of Ni(abt)_2_ crystal, indicating that Ni(abt)_2_ is weakly coupling with MoS_2_ surface. Moreover, the distance between MoS_2_ and Ni(abt)_2_ is above 3.0 Å (a typical distance via weak interaction), further suggesting that Ni(abt)_2_ is physically self‐assembled on MoS_2_ surface via intermolecular weak interaction and thus will not bring damage on MoS_2_. Therefore, the self‐assembly of Ni(abt)_2_ molecules on MoS_2_ keeps high HER activities of both N sites from on‐MoS_2_ Ni(abt)_2_ and edge sites from MoS_2_. The coverage is further increased by two times, close to the coverage limit. Under such a high coverage, the N site still owns high HER activity and is considerably more superior than S and Ni sites (see Figure S2, Supporting Information), in good agreement with the results in Figure 1e, showing that the system is insensitive to the coverage.

### 2.2 Stability of On‐MoS_2_ Ni(abt)_2_

**Figure 2:**
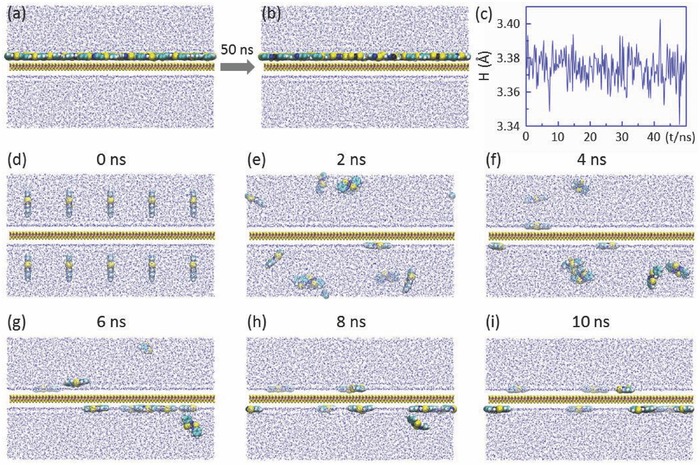
a) Initial structure of on‐MoS_2_ Ni(abt)_2_ molecules in water. b) Evolution of (a) after 50 ns. c) Average height of Ni(abt)_2_ molecules relative to MoS_2_ as a function of time. d–i) Dynamic process of Ni(abt)_2_ molecules from staying in water to lying on MoS_2_. The snapshots were taken every 2 ns.

Another essential prerequisite is that Ni(abt)_2_ molecules physically self‐assembled on MoS_2_ via intermolecular weak interactions are stable on MoS_2_ and will not diffuse into water. **Figure** **2**a depicts the initial structure of Ni(abt)_2_ molecules on MoS_2_ placed in water and Figure 2b presents the structure after 50 ns. From Figure 2b, it can be seen that all Ni(abt)_2_ molecules still adhere to the MoS_2_ surface and do not diffuse into water. The average height of Ni(abt)_2_ molecules relative to the MoS_2_ substrate is recorded in Figure 2c. The height only fluctuates in a tiny range (less than 0.1 Å), further demonstrating that on‐MoS_2_ Ni(abt)_2_ molecules are very stable in water. Like the case at 300 K, the system is still very stable even at a slightly higher temperature, e.g., 350 K (see Figure S3, Supporting Information). We also explore the opposite case, that is Ni(abt)_2_ molecules initially placed in water (see Figure 2d). Figure 2e–i records the evolution of the structure in Figure 2d with time. Obviously, the molecules fast escape from water and move onto the MoS_2_ surface, which unambiguously illustrates that Ni(abt)_2_ prefers staying on the surface of MoS_2_ rather than diffusing into water.

H_2_ is generated from large‐area in‐plane domains of MoS_2_, so it is desirable to explore the effect of H_2_ on the system stability. Figure S4 of the Supporting Information records the dynamic evolution of 80 Ni(abt)_2_ and 20 H_2_ molecules placed on MoS_2_. H_2_ molecules fast escape from MoS_2_ and diffuse into water, showing that on‐MoS_2_ H_2_ is very unstable. The intrinsic reason is owing to the extremely weak interaction between H_2_ and MoS_2_, only 0.06 eV. By contrast, Ni(abt)_2_ molecules have not been influenced by H_2_ and adhere to the MoS_2_ surface throughout. Therefore, we conclude that the generated H_2_ fast diffuses into water and does not give rise to the instability of Ni(abt)_2_ on MoS_2_.

### 2.3 Preparation of On‐MoS_2_ Ni(abt)_2_

We have demonstrated that on‐MoS_2_ Ni(abt)_2_ is of high HER activity above. Next, we will explore how to prepare self‐assembled Ni(abt)_2_ monolayers on MoS_2_. Many studies have shown that ultrathin self‐assembled organic monolayers can be prepared through a facile liquid‐deposition method,^53^, ^54^, ^55^, ^56^, ^57^ i.e., organic molecules are first dissolved in solvent and self‐assembled organic monolayers can be naturally generated on the substrate on which the mixed solution is dropped. From the preparation process, two key points toward the preparation of self‐assembled Ni(abt)_2_ monolayers on MoS_2_ can be derived: (i) an appropriate solvent that can dissolve Ni(abt)_2_ and (ii) dissolved Ni(abt)_2_ molecules that can deposit onto MoS_2_.

**Figure 3:**
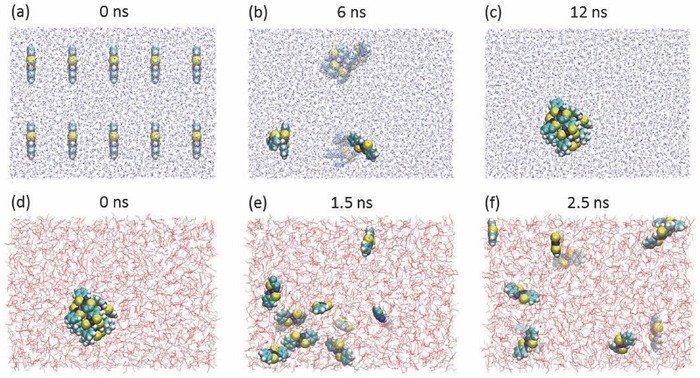
Dynamic behaviors of Ni(abt)_2_ in a–c) water and d–f) diethyl ether. a) Initial structure of Ni(abt)_2_ molecules in water and b,c) snapshots taken from the evolution of a) 6 and 12 ns later. d) Initial structure of the Ni(abt)_2_ cluster formed in c) placed in diethyl ether. e,f) Snapshots at 1.5 and 2.5 ns taken from the evolution of (d). Note that the hydrogen atoms of diethyl ether are not displayed for better visual effect.

**Figure** **3**a–c and Movie S1 (Supporting Information) describe the dynamic behavior of Ni(abt)_2_ molecules in water. Ni(abt)_2_ molecules are uniformly placed in water in the beginning (Figure 3a); thereafter, Ni(abt)_2_ molecules gradually aggregate (Movie S1, Supporting Information) till 12 ns at which all Ni(abt)_2_ molecules aggregate together as shown in Figure 3c. The results show that Ni(abt)_2_ is insoluble in water, in line with the experiment.^45^ Alternatively, Ni(abt)_2_ is known to be crystallized from diethyl ether,^45^ so MD simulation of Ni(abt)_2_ molecules in diethyl ether is performed. As expected, the Ni(abt)_2_ molecules are fast separated from each other in diethyl ether (see Figure 3d–f; Movie S2, Supporting Information) instead of aggregation as found in water, indicating that Ni(abt)_2_ molecules can be dissolved in diethyl ether well.

**Figure 4:**
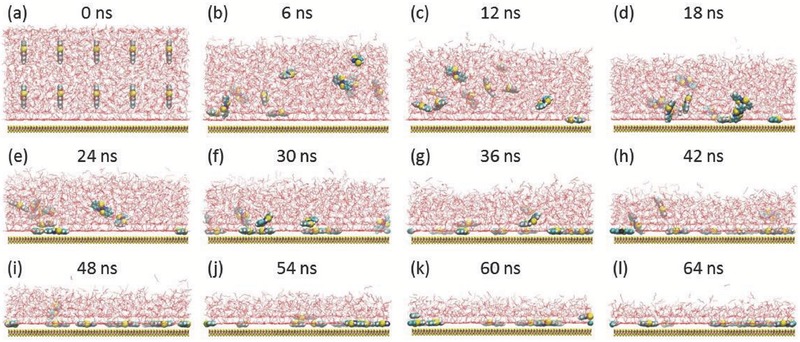
Liquid deposition of Ni(abt)_2_ molecules in diethyl ether solution onto MoS_2_ with the volatilization of diethyl ether molecules. a) Initial structure and b–l) snapshots taken from the evolution of (a) after 6, 12, 18, 24, 30, 36, 42, 48, 54, 60, and 64 ns, respectively. For better visual effect, the hydrogen atoms of diethyl ether are not displayed.

Next, we investigate whether Ni(abt)_2_ molecules in diethyl ether can deposit onto MoS_2_. Note that diethyl ether is highly volatile if exposed to air, so diethyl ether molecules must gradually diffuse into air when Ni(abt)_2_ solution is dropped on MoS_2_. Therefore, to reflect the actual situation more reasonably, the volatilization process is considered in our simulations. **Figure** **4**a presents the initial structure of Ni(abt)_2_ solution dropped onto MoS_2_. Obviously, diethyl ether molecules gradually escape from the solution, but Ni(abt)_2_ molecules remain in solution and gradually deposit onto MoS_2_ (see Figure 4b–l). Finally, all Ni(abt)_2_ molecules deposit onto MoS_2_ as shown in Figure 4l, showing clearly that self‐assembled Ni(abt)_2_ monolayers on MoS_2_ can be prepared through a simple liquid‐deposition method.

### 2.4 Bifunctional Catalysts for Overall Water Splitting

**Figure 5:**
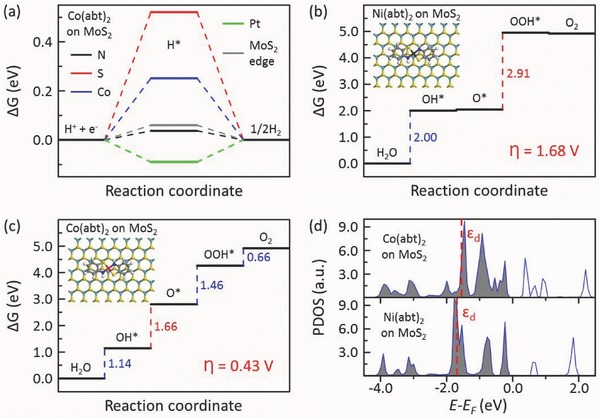
a) Calculated Δ*G* profile of HER at N, S, and Co sites of on‐MoS_2_ Co(abt)_2_. Δ*G* diagrams for OER at b) the Ni site of on‐MoS_2_ Ni(abt)_2_ and c) the Co site of on‐MoS_2_ Co(abt)_2_. d) Calculated projected density of states (PDOS) of d band for the Ni atom of Ni(abt)_2_ on MoS_2_ and the Co atom of Co(abt)_2_ on MoS_2_. The d band center is marked by the red dashed line and the Fermi level is set as zero.

We have shown that the on‐surface self‐assembly of Ni(abt)_2_ retains the merits of high HER activities from both MoS_2_ and Ni(abt)_2_. Also, the integration of various active sites from components of the complex is promising for bifunctional or multifunctional catalysts, as learned from nanoscale alloys^37^, ^38^, ^39^, ^40^, ^41^ and doping carbon‐based materials,^42^, ^43^, ^44^ which are common approaches toward the preparation of bifunctional catalysts for overall water splitting. Hence, we want to explore whether on‐surface self‐assembly has potential to be a new strategy to prepare bifunctional catalysts. The self‐assembled system consisting of MoS_2_ and Ni(abt)_2_ has been demonstrated to be of high HER activity, so if on‐MoS_2_ Ni(abt)_2_ is also of high OER activity, it can be served as the bifunctional catalyst for overall water splitting. For OER, the reaction consists of four elementary processes

(2) $$H_{2}O\left(1\right)\;+\;*\;⇌\;*\mathrm{OH}\;+\;H^{+}\;+\;e^{-}$$

(3) $$\mathrm{OH}*\;⇌\;*O\;+\;H^{+}\;+\;e^{-}$$

(4) $$*O\;+\;H_{2}O(l)\;⇌\;*\mathrm{OOH}\;+\;H^{+}\;+\;e^{-}$$

(5) $$*\mathrm{OOH}\;⇌\;*\;+\;O_{2}(g)\;+\;H^{+}\;+\;e^{-}$$

where the * represents the catalytic site. The smaller the overpotential η is, the higher the OER activity is. η is defined as

(6) $$\eta \;=\;\max\;\left[\Delta G_{1},\Delta G_{2},\Delta G_{3},\Delta G_{4}\right]\mathrm{/e}\;-\;1.23\;V$$

in which Δ*G*_1_, Δ*G*_2_, Δ*G*_3_, and Δ*G*_4_ are the free energy differences of the four processes mentioned above, respectively. In general, oxygen evolution or reduction occurs at transition metal atoms. As shown in **Figure** **5**b, for the Ni site of on‐MoS_2_ Ni(abt)_2_ the calculated Δ*G*_1_, Δ*G*_2_, Δ*G*_3_, and Δ*G*_4_ are 2.00, 0.04, 2.91, and −0.03 eV, respectively. The rather positive Δ*G*_1_ and Δ*G*_3_ suggest the weak bonding strength between the Ni site and OH* and OOH*. The process from O* to OOH* is the potential‐determining step and the overpotential η reaches up to 1.68 V, showing that the OER activity is very poor.

As Co‐based materials are another commonly applied and nonprecious electrocatalysts, we further investigate the catalytic behavior of on‐MoS_2_ Co(abt)_2_. Like Ni(abt)_2_ for HER, the N site owns high HER activity (Δ*G* is only 0.04 eV) and its HER activity is comparable to that of on‐MoS_2_ Ni(abt)_2_ (see Figure 5a,e). Figure S5 of the Supporting Information further demonstrates that Co(abt)_2_ molecules on MoS_2_ are very stable in water and they prefer staying on MoS_2_ instead of in water as well. The high HER activity and water stability guarantee that self‐assembled Co(abt)_2_ monolayers can be used to effectively utilize the large‐area basal plane of MoS_2_ for HER. In addition, self‐assembled Co(abt)_2_ monolayers on MoS_2_ can also be prepared by a simple operation similar to on‐MoS_2_ Ni(abt)_2_, as shown in Figure S6 of the Supporting Information. Unlike Ni(abt)_2_ for OER, each step is moderate for the Co site of on‐MoS_2_ Co(abt)_2_ (see Figure 5c). The potential‐determining step is the process from OH* to O* and the overpotential η is only 0.43 V, comparable to traditional precious metal‐based OER catalysts in a range of about 0.3–0.7 V,^51^, ^58^, ^59^ showing that OER can be efficiently catalyzed at the Co site of on‐MoS_2_ Co(abt)_2_. Our calculations show that OER can be catalyzed by Co(abt)_2_ along the four‐electron pathway. However, this does not mean that OER is impossible to occur at Co(abt)_2_ along other pathways. There may even exist better pathways with lower OER overpotentials compared to the four‐electron pathway, but this will not change our conclusion that Co(abt)_2_ can effectively catalyze OER. Therefore, we can conclude that on‐MoS_2_ Co(abt)_2_ is of both high HER and OER activities, suggesting that on‐surface self‐assembly can serve for the preparation of efficient bifunctional catalysts toward overall water splitting.

The binding energies of MoS_2_ and the intermediates of M(abt)_2_ during HER and OER (see Figure S7a–e, Supporting Information) are −1.76, −1.59, −1.56, −1.43, and −1.75 eV, respectively, which is comparable to that between MoS_2_ and M(abt)_2_ (−1.63 eV). This shows that M(abt)_2_ on MoS_2_ is still stable during HER and OER. The distinct catalytic performance for the Ni site of Ni(abt)_2_ and the Co site of Co(abt)_2_ can be understood well by the theory of d band center (ε_d_). The closer to the Fermi level the ε_d_ is, the stronger the binding of the adsorbate and the catalytic site is. As shown in Figure 5d, ε_d_ of the Co atom is closer to the Fermi level; therefore the binding of the adsorbates and the Co atom is stronger, leading to lower Δ*G* of the adsorbate H, OH, and OOH (see Figures 1e and 5a–c). However, the adsorbate O is abnormal whose binding with Co(abt)_2_ is much weaker. Such an abnormal phenomenon can be explained by the structure difference. As opposite to the other cases, the O atom is unable to steadily adsorb on the Ni atom and it insets into the Ni—S bond (see Figure S7, Supporting Information), which strengthens the bonding of the O atom and Ni(abt)_2_, thus resulting in a lower Δ*G*.

## 3 Conclusion

In summary, we have reported a new and promising avenue (on‐surface self‐assembly) to make full use of the large‐area in‐plane domains of MoS_2_ for HER and prepare bifunctional catalysts for overall water splitting. We demonstrated that the weak interaction between M(abt)_2_ molecules and MoS_2_ plays an important role in the effective bottom‐up on‐surface self‐assembly from three aspects: (a) rendering a facile preparation by liquid‐deposition method possible, (b) keeping M(abt)_2_ molecules distributing on the MoS_2_ surface instead of diffusing into water, and (c) preserving high HER activities of both edge sites of MoS_2_ and N sites of M(abt)_2_ molecules. Therefore, efficient utilization of the large‐area basal plane of MoS_2_ for HER is achieved based on significantly increased active sites by on‐surface self‐assembly, which is also anticipated to be generally applicable to exploit the basal plane of other transition metal dichalcogenides. In addition, the bottom‐up self‐assembly of molecules onto MoS_2_ surface can bring other desired properties for multifunctional applications. For example, our results show that on‐MoS_2_ Co(abt)_2_ is able to catalyze OER, suggesting that on‐surface self‐assembly can also be used to prepare bifunctional catalysts for overall water splitting. This work provides an effective and facile strategy, on‐surface self‐assembly, to prepare efficient catalysts for water splitting, thereby offering high possibility toward achieving a sustainable energy supply.

## 4 Experimental Section

*DFT Calculations*: All DFT calculations were carried out through the projector augmented wave method^60^ with spin polarization and van der Waals (vdW) modification (D3)^61^ as implemented in the Vienna ab initio simulation package.^62^ The exchange‐correlation functional was built on the functional of Perdew, Burke, and Ernzerhof of generalized gradient approximation.^63^ A 7 × 3√3 × 1 supercell was adopted for the MoS_2_ substrate. The Brillouin zone was sampled using the Monkhorst–Pack scheme with k‐point mesh of 3 × 3 × 1 for 2D systems and 3 × 3 × 3 for 3D systems. The kinetic energy cutoff for the plane‐wave basis set was set as 500 eV. All structures were fully relaxed until reaching the convergence threshold of 0.02 eV Å^−1^ for force and 10^−4^ eV for energy.

*MD Simulations*: All simulations were performed by using the software Gromacs version 4^64^ under the condition of 300 K. Intermolecular interactions consist of the vdW and electrostatic interactions, which were calculated according to Coulomb's law and 12–6 Lennard‐Jones (LJ) potential, respectively. The cutoff distance of intermolecular interactions was set to 1.5 nm outside which the smoothed particle mesh Ewald sum^65^ was used to deal with the long‐range electrostatic interaction and the vdW interaction was not considered. The time step was set to 1 fs and the berendsen thermostat and barostat were utilized to control temperature and pressure, respectively. Periodic boundary conditions were employed to avoid the edge effect. Force field (FF) parameters of Ni(abt)_2_ or Co(abt)_2_ were constructed by combining all‐atom Amber99sb FF^66^, ^67^ with universal FF.^68^ The reliability of the constructed FF parameters is guaranteed by the good agreement between simulation and experiment for the lattice parameters of Ni(abt)_2_ crystal (see Table S1). Moreover, the simulated dynamic behavior of Ni(abt)_2_ in water and diethyl ether, i.e., insoluble in water and soluble in diethyl ether, agrees with the experimental results, further guaranteeing the reliability of the constructed FF parameters. The FF parameters for diethyl ether were built on all‐atom Amber99sb FF and water was described by extended simple point charge model. Molecular partial charges were obtained based on the Chelpg methodology.^69^ The atomic charges of MoS_2_ were obtained from ref. 70 and its LJ parameters were derived according to the calculated molecule‐MoS_2_ interaction from DFT calculations. The size of MoS_2_ used in simulations is 10.18592 nm × 9.92124 nm consisting of 1152 Mo and 2304 S atoms. The evaporation process of diethyl ether in Figure 4 was mimicked by exhausting the escaped molecules every 2 ns.

*ΔG Calculations*: Δ*G* is the difference of the free energy (*G*) between products and reactants and *G* is calculated as

(7) $$G\;=\;E\;+\;E_{\mathrm{ZPE}}\;-\;TS$$

where *E*, *E*_ZPE_, *T*, and *S* represent the energy, the zero‐point energy, the reaction temperature, and the entropy, respectively. The difference of the zero‐point energies is obtained via vibrational frequency calculation for the catalyst with and without adsorbed species. The difference between the entropies of the catalyst with and without adsorbed species is very small and neglected. All calculations were done under the standard hydrogen electrode in which the free energy of protons and electrons (H^+^ + *e* ^−^) is taken as a half of the free energy of gas H_2_. The solvation corrections to account for the effect of water as well as the zero‐point energies and the entropies of gas H_2_ and liquid H_2_O were obtained from ref. 49. The free energy of gas O_2_ ($G_{O_{2}(g)}$) is calculated by

(8) $$G_{O_{2}(g)}\;=\;4.92\;\mathrm{eV}\;+\;2G_{H_{2}O(l)}\;-\;2G_{H_{2}(g)}$$

in which $G_{H_{2}(g)}$ represents the free energy of gas H_2_, $G_{H_{2}O(l)}$ stands for the free energy of liquid H_2_O, and 4.92 eV is the reaction free energy for splitting liquid H_2_O into gas H_2_ and O_2_.

## Conflict of Interest

The authors declare no conflict of interest.

## Supporting information

Supplementary

Supplementary

Supplementary
