# Supplementary material for: Exploitation of the Large‐Area Basal Plane of MoS2 and Preparation of Bifunctional Catalysts through On‐Surface Self‐Assembly
Source: Adv Sci (Weinh). 2017 Sep 23;4(12):1700356. doi: 10.1002/advs.201700356 (PMC5737238; doi:10.1002/advs.201700356)
Supplement: Supplementary file 1 — Supplementary [file ADVS-4-na-s001.pdf]

Copyright WILEY-VCH Verlag GmbH & Co. KGaA, 69469 Weinheim, Germany, 2017.

ADVANCED SCIENCE
Open Access

Supporting Information

for *Adv. Sci.,* DOI: 10.1002/advs.201700356

Exploitation of the Large-Area Basal Plane of MoS_2 and Preparation of Bifunctional Catalysts through On-Surface Self-Assembly

*Yinghe Zhao, Qiang Li, Li Shi, and Jinlan Wang**

Copyright WILEY-VCH Verlag GmbH & Co. KGaA, 69469 Weinheim, Germany, 2016.

Supporting Information

Exploitation of the Large-Area Basal Plane of MoS_2 and Preparation of Bifunctional Catalysts through On-Surface Self-Assembly

*Yinghe Zhao, Qiang Li, Li Shi, and Jinlan Wang**

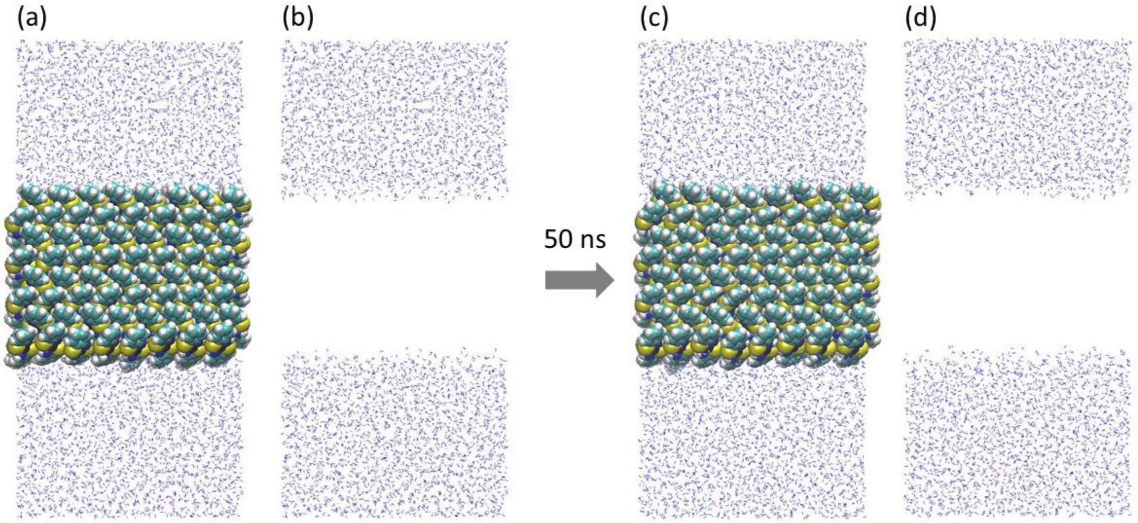

Figure S1. (a) Initial structure of $Ni(abt)_2$ crystal placed in water and (b) water distribution of the structure in (a). (c) Evolution of (a) 50 ns later and (d) water distribution of the structure in (c).

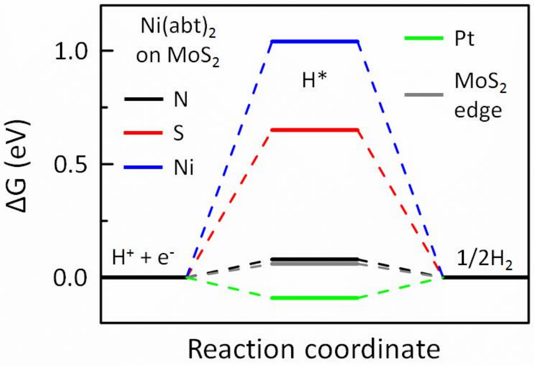

Figure S2. Calculated ΔG profile of HER at N, S, and Ni sites of on-MoS_2 $Ni(abt)_2$ with doubling the coverage in Figure 1c.

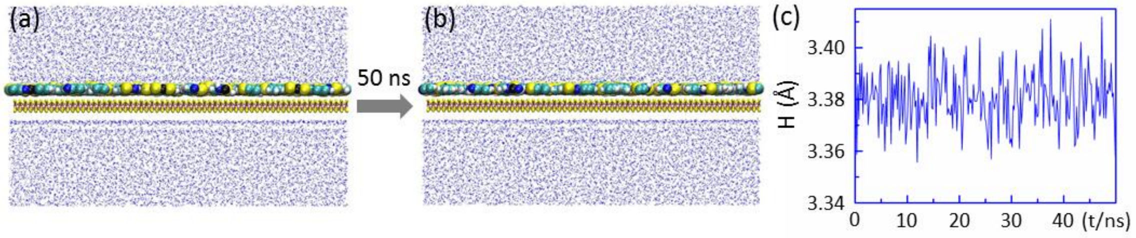

Figure S3. (a) Initial structure of on-MoS_2 $Ni(abt)_2$ molecules in water. (b) Evolution of (a) after 50 ns at 350 K. (c) Average height of $Ni(abt)_2$ molecules relative to MoS_2 as a function of time.

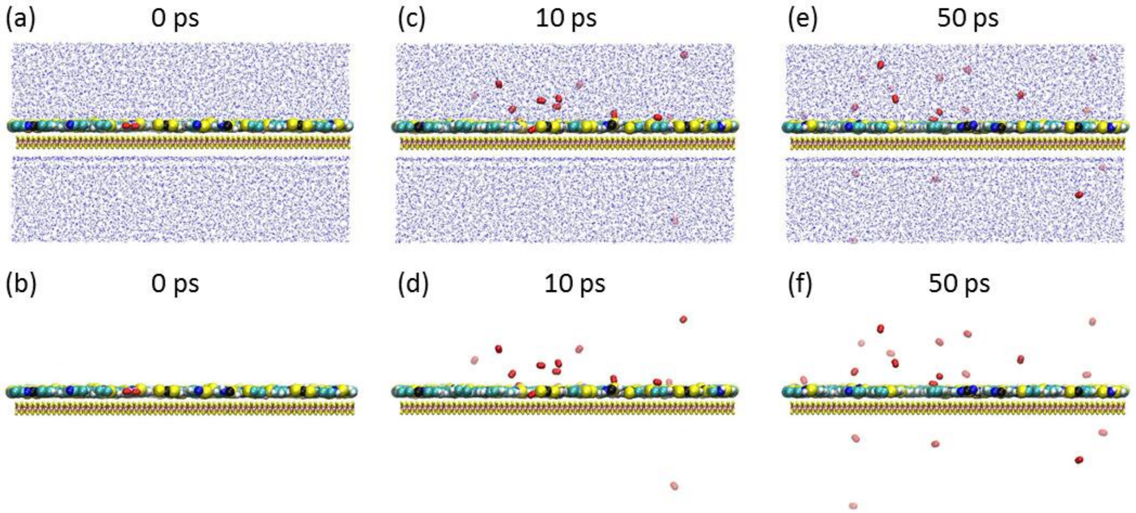

Figure S4. (a) Initial structure of 80 $Ni(abt)_2$ and 20 H_2 molecules (red balls) on MoS_2. Note that water molecules are screened for better visual effect and the structure without water is presented in (b). (c-f) Evolutions of (a) after 10 ps (c,d) and 50 ps (e,f).

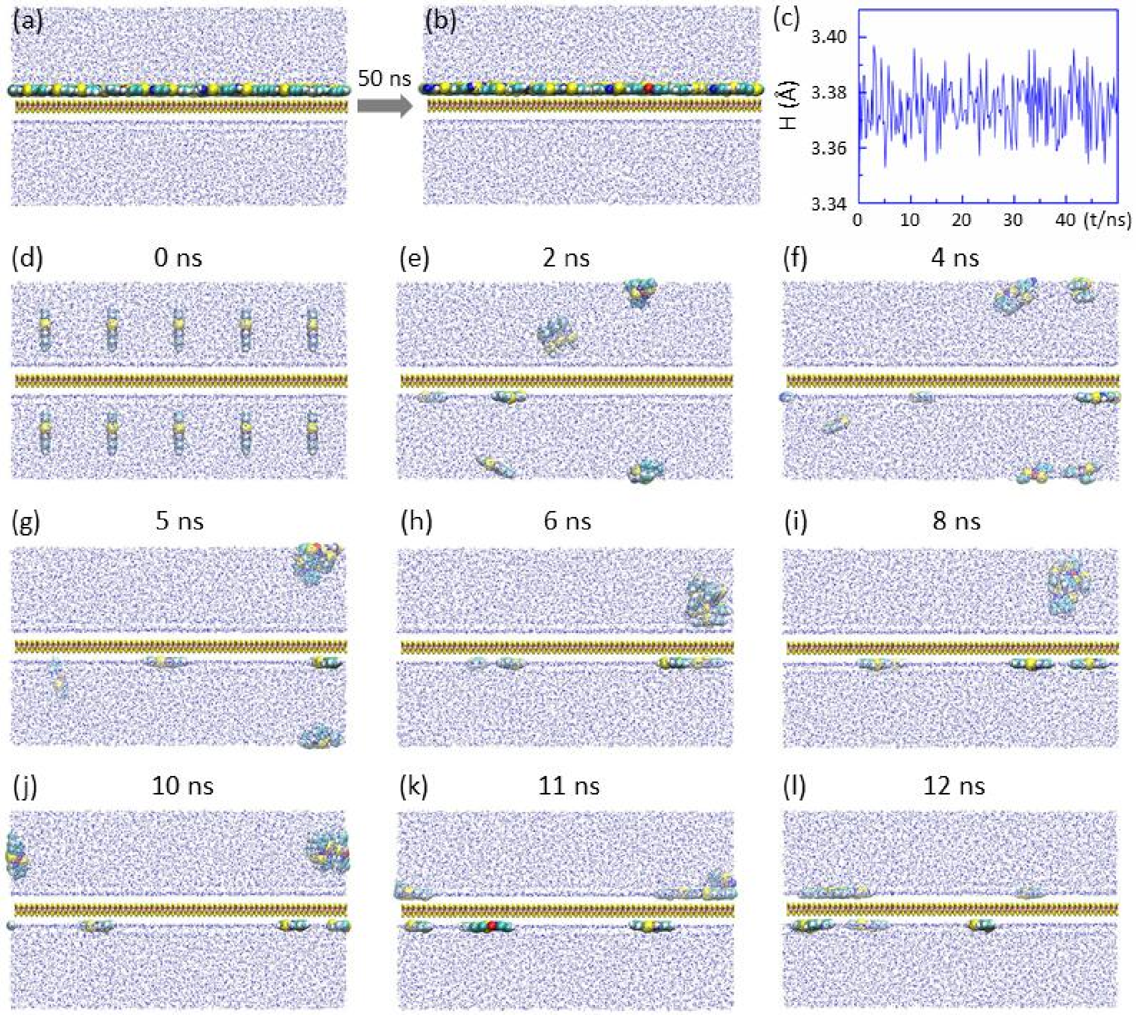

Figure S5. (a) Initial structure of on-MoS_2 $Co(abt)_2$ molecules in water. (b) Evolution of (a) after 50 ns. (c) Average height of $Co(abt)_2$ molecules relative to MoS_2 as a function of time. (d-l) Dynamic process of $Co(abt)_2$ molecules from staying in water to lying on MoS_2. The initial structure is recorded in (d) and the snapshots taken at t = 2, 4, 5, 6, 8, 10, 11, and 12 ns are presented in (e-l).

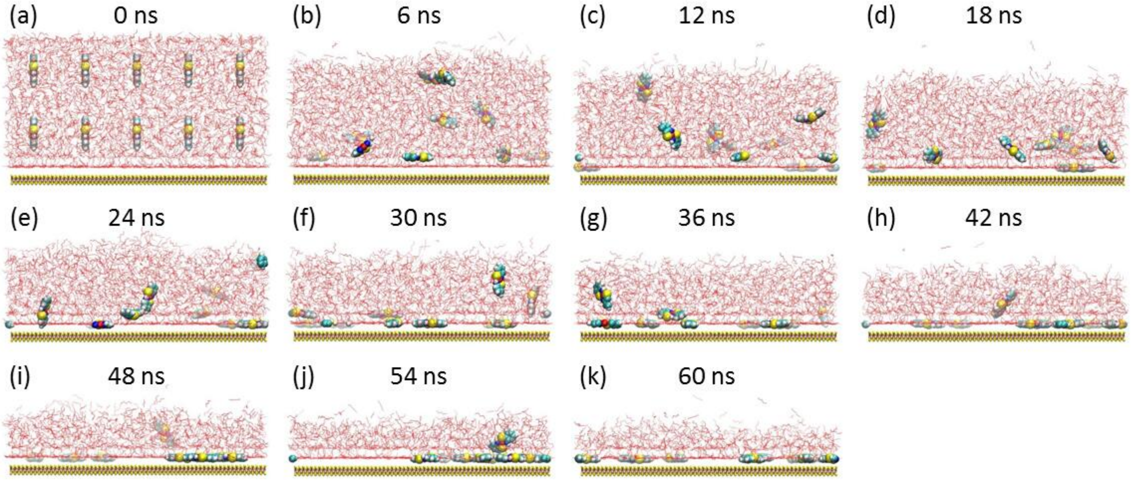

Figure S6. Liquid deposition of $Co(abt)_2$ molecules on MoS_2 with the volatilization of diethyl ether molecules. (a) Initial structure and (b-k) snapshots taken from the evolution of (a) every 6 ns. For better visual effect, the hydrogen atoms of diethyl ether are not displayed.

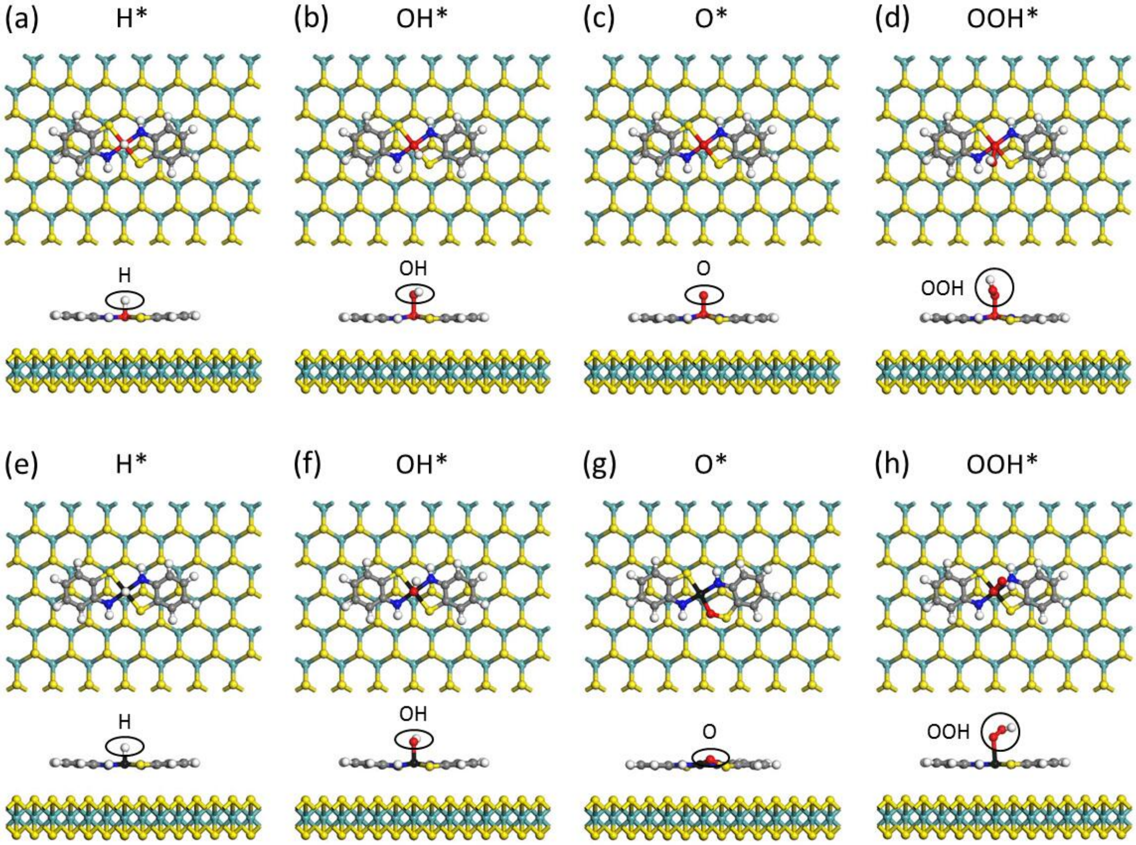

Figure S7. Structures of adsorbates H, OH, O, and OOH on on-MoS_2 $Co(abt)_2$ (a-d) and $Ni(abt)_2$ (e-h).

Table S1. Comparison of the calculated $Ni(abt)_2$ lattice parameters through MD simulations and the experimental results.

	$Ni(abt)_2$ lattice parameters		
	a (nm)	*b* (nm)	*c* (nm)
MD[a]	1.233	0.590	0.816
Exp[b]	1.230	0.588	0.810

[a]MD represents the simulation results; [b]Exp stands for the results from experiment.[1]

Reference

[1] A. Das, Z. Han, W. W. Brennessel, P. L. Holland, R. Eisenberg, *ACS Catal.* **2015**, *5*, 1397.
